# The pitfalls of electronic health orders: development of an enhanced institutional protocol after a preventable patient death

**DOI:** 10.1186/s13037-014-0039-0

**Published:** 2014-09-27

**Authors:** Brandon J Manley, Rebecca K Gericke, John A Brockman, Jennifer Robles, Valary T Raup, Sam B Bhayani

**Affiliations:** Division of Urology, Department of Surgery, Washington University in St. Louis, St. Louis, MO USA; Division of Urologic Surgery, Washington University School of Medicine, 4960 Children’s Pl., Campus, Box 8242, St. Louis, MO 63110 USA

**Keywords:** MeSH, Hematuria, Communication, Delivery of health care, Urinary bladder, Consultants

## Abstract

**Background:**

Continuous bladder irrigation (CBI) is a long-standing treatment used in the setting of gross hematuria and other acute bladder issues. Its use has traditionally been reserved for patients under direct urologic care, but with the constraints of modern large-hospital healthcare, many patients have CBI administered by providers unfamiliar with its use and potential complications.

**Findings:**

There were 136 CBI orders placed in 2013 by non-urologic providers. The biggest hazard found in our analysis was the requirement for entering a rate of irrigation administration. Nurses with no experience with CBI viewed this order as an indication to administer via an infusion pump, which can easily exceed the mechanical integrity of the bladder and increase the risk of bladder perforation. Our panel also found that due to lack of experience by nurses and non-urologic providers, that signs and symptoms of CBI dysfunction were not common knowledge. Also we found that non-urologic providers were unfamiliar with administration and dosing of medications for CBI patients to help with the intrinsic discomfort with CBI administration.

**Conclusions:**

In our revised order set we found that removing the requirement for an infusion rate, along with placing warnings in the CPOE, helped staff better understand this possible complication. We created a best practice alert in our CPOE to strongly recommend the urology service be consulted. Communication text boxes were added to the order set to help staff be aware of the signs and symptoms of CBI dysfunction, along with a guide for trouble shooting.

## Background

Continuous bladder irrigation (CBI) is a long-standing treatment commonly used in the setting of gross hematuria [1]. Other uses for CBI can increasingly be found in non-urologic patients, such as for with hemorrhagic cystitis after hematopoietic stem cell transplantation or candiduria in intensive care unit patients [2,3]. The process of administering CBI involves the use of a three-way catheter placed into the bladder and connected to large volume bags of normal saline. This provides continuous irrigation into, and out of, the bladder. This allows blood from the genitourinary tract to be immediately evacuated, thus preventing clots from forming. The goal of this treatment is to prevent the need for surgical intervention by continuously flushing out clots, while the bleeding area heals.

The administration of CBI requires intensive nursing support for proper administration and appropriate physician ordering for safety. During a 12 month period at a large urban academic hospital (1,305 beds), 136 CBI orders were entered by non-urology care providers. This increasing use of CBI by staff not aware of the physics [4,5] of irrigation and unfamiliar with the risk factors [6] or management of CBI complications creates a scenario for possible adverse events. In particular, inflow of irrigant should be done via low gravity, and not forced into the bladder with pumps or pressure. This allows the irrigant to stop immediately if the outflow is compromised or clotted. If the irrigant is forced into the bladder with an infusion pump or manual irrigation while the outflow is clotted, the contents can contribute to, or exacerbate bladder rupture.

The origin of our study arose after a sentinel event in which a patient suffered iatrogenic intraperitoneal bladder rupture while being administered CBI by providers unfamiliar with its usage. While critically ill and mechanically ventilated, he developed intermittent gross hematuria. CBI was started by the intensive care team; however, instead of instilling the irrigation via low gravity, they employed the use of an infusion pump, similar to that used for infusing intravenous fluids. Twenty-four hours after the CBI was restarted, the patient developed a bladder rupture that was attributed to the high pressure with which the irrigation fluid was infused.

## Methods

An interdisciplinary team of physicians, nurses and pharmacists was constructed to initiate a root-cause analysis after the event. The study was approved by the Washington University in St. Louis Human Research Protection Office (ID: 201403143). The goal of the initial meeting was to obtain feedback from staff members inexperienced with CBI about their understanding of CBI, its techniques, and the management of complications. Staff directly involved with the sentinel event and others not involved were interviewed. Included in these discussions were: nurses who care for and monitor patients receiving CBI, physicians who order medications and direct the care of such patients, and pharmacists who supply irrigation fluids and medications used in patients being treated with CBI.

A team from the computerized physician order entry (CPOE) and electronic health record (EHR) system was also involved to critically review the current CBI order set used in the hospital. Each step of the ordering process was reviewed.

The authors initially examined the 136 CBI orders placed by non-urology staff over the prior 12 months, scrutinizing discrepancies between these orders and those commonly used by urologic providers. This information was used to direct specific questions to the staff and nursing about how these orders were perceived and instituted with regards to non-urologic patients on CBI.

## Findings

The investigation revealed several systemic weaknesses in the care and management of patients undergoing CBI. The weaknesses could be categorized into CPOE/EHR flaws (the requirement to enter a rate of CBI, which would necessitate a pump to measure, rather than hanging it to gravity), and a lack of oversight via urological consultation. Clinicians and staff with long standing experience in managing CBI patients, along with input from those who managed and constructed the hospitals EHR/CPOE, identified several areas for improvement.

Irrigations infused with continuous force can easily exceed the mechanical integrity of the bladder and increase the risk of bladder rupture. The EHR order could not be completed without entering in a rate, and only numeric values were accepted. In discussing this issue with pharmacy and the computer support team, it was found that when the original order-set was created the irrigation for CBI has been documented and billed as an intravenous fluid (IVF); this lead the computer team to believe the ordering system for CBI irrigation should be similar to all other IVF’s, which require a rate to be entered. After identifying this issue, the panel decided that the previous order-set had to be modified. First, the numeric rate of irrigation was removed all together, and instead, instructions for administration by gravity along with warnings against the use of an infusion pump were included (Table 1).

**Table 1:** **Continuous bladder irrigation order**

**Order name**	**Volume**	**Route**	**Rate**	**Indication**	**Comments**
☐ Normal Saline for Continuous Bladder Irrigation	3000 ml	CBI	Gravity: hang to gravity and titrate for clear, clot free urine	(to be filled in by MD)	WARNING: Run to gravity only. Do not place on pump. Titrate to keep urine clear and clot free. Monitor closely, recording hourly I &O. If catheter stops draining or slows, immediately stop CBI and call Urology & House Officer. Attempt to irrigate once with 60 ml NS.

CBI = continuos bladder irrigation, I&O = Intake and output of fluid, NS = normal saline.

In addition, further discussions with the nursing staff revealed that there was some confusion about how to order CBI supplies and how to keep up with the frequency of irrigation bag changes. Several orders were included within the new CBI order set to aid nurses in locating supplies; also provided were numbers to call if these supplies were unavailable (Table 2). Furthermore, the number of irrigation bags sent to the patient’s room at the time of the initial order (usually two 3 L bags) was increased to reduce the chance irrigation would be interrupted. The pharmacy also made changes so that several bags were reserved ahead of time for each patient undergoing CBI.

**Table 2:** **Supplies needed at bedside**

**Supply order name**	**Additional information**
☒ Large Foley Bag	Call Stores (#), if not floor stocked
☒ Large bore Three way catheter	Call Stores (#), if not floor stocked
☒ IV pole	No IV pump, Pole only.
☒ Large Fluid Bags	From Pharmacy

IV = intravenous.

The panel also found that that the signs and symptoms of CBI malfunction were not common knowledge amongst non-urologic nurses and physicians. Previously, no directions were included in the order-set regarding side effects and symptoms of CBI malfunction. There also was no trouble shooting directions. Signs and symptoms of CBI malfunction, including urine leaking around the catheter and suprapubic distention were added to the comprehensive CBI order set. The new order set includes several nursing communication orders to aid in management (Table 3) and monitoring (Table 4) of patients undergoing CBI. Better communication will aid in earlier detection of complications and hopefully prevent sentinel events from occurring.

**Table 3:** **When to notify a physician**

**Order name**	**Notify**	**Urine output**	**Additional information**
☒	House Officer & Urology	If urine output decreases, or stops.	First, stop infusion, then notify HO & urology.
☒	House Officer & Urology		Prior to discontinuation of CBI
☒	House Officer & Urology		For any interruption of continuous flow of urine into the bladder or into the drainage bag
☒	House Officer & Urology		For patients complaints of suprapubic pain or discomfort
☒	House Officer & Urology		If unable to flush or if irrigating more than once every 4 hours.

HO = house officer, CBI = continuous bladder irrigation.

**Table 4:** **Nursing/treatments**

**Order name**	**Frequency**	**Additional information**
☒ Titrate fluid to keep urine clear	PRN	WARNING:
☒ Irrigate/aspirate every 4 hours as needed with 60 ml normal saline. Repeat as needed.	Q 4 hours PRN	Notify HO and Urology if unable to aspirate/irrigate or if frequency is less than every 4 hours.
☒ Do not let irrigation fluid run out establish and maintain supply of fluid through communication with pharmacy	Continuous through treatment	Establish supply of irrigation fluid from pharmacy using communication order.
☒ If urine clear for 24 hours, notify physicians for possible discontinuation of CBI	PRN	CALL UROLOGY!

PRN = as needed, HO = house officer, CBI = continuous bladder irrigation.

The panel also recognized that non-urologic providers were unfamiliar with the administration of medications commonly used to help alleviate the discomfort caused by CBI. Therefore, several medications were introduced within the EHR order set for CBI. These orders included commonly used dosing and frequency for medication like oxybutynin, hycosamine and belladonna/opium suppositories.

**Figure 1:**
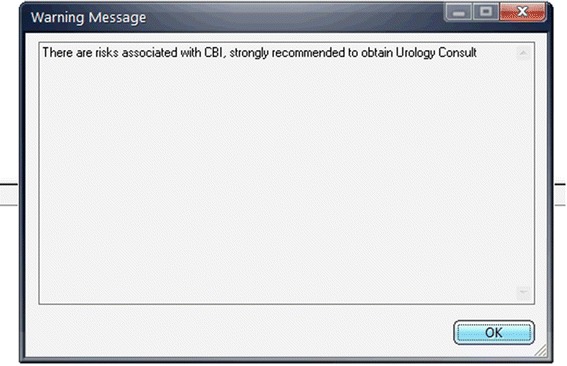
**Screenshot of best practice alert icon placed in CPOE at initiation of revised CBI order set.**

The second major corrective action was to strongly recommend urological consultation. Although not required for the CBI order, a best practice alert (Figure 1) was created in the CPOE/EHR system when the order for CBI was entered. The soft stop consisted of a popup suggesting urological consultation and a pager number was provided.

## Conclusions

In the revised order set, a best practice alert strongly recommending a urologic consultation was implemented and the CBI infusion rate requirement was removed in order to help non-urologic physicians and nurses with safe CBI ordering and administration.

The revision of our CBI electronic order set highlights several issues present in today’s medical environment. While the use of CPOE has made for a more efficient and effective method for the delivery of patient care, our investigation revealed the continued need to have clinician oversight and scheduled review in the development of these orders. While no clinician would prefer to learn of mistakes after a patient has a complication we were pleased to see the effective solutions brought forth from our investigation and our patient safety review committee. The involvement of not only the clinicians but also the nursing staff, pharmacists and computer technologists helped bring many of the barriers to patient safety to light and also lead to the comprehensive changes described in our study. The fostering of collaboration should not be limited to the clinicians when it comes to improving patient care and the benefits from involvement of all levels for those involved with patients care should be highly encouraged.

## Abbreviations

CBI: Continuous bladder irrigation
CPOE: Computerized physician order entry
EHR: Electronic health record
HO: House officer
